# Parliament and the Profession

**Published:** 1917-12-29

**Authors:** 


### Parliament and the Profession.

### Grants to Auxiliary Hospitals.

THE Financial Secretary to the War Office informed Sir William Collins that grants to large voluntary hospitals, such as the London Hospital and others, are under review, and that the question of increasing the grant is being considered.

### Military Hospital at Canterbury.

MR. HIGHAM, in a question to the Under-Secretary of State for War, called attention to the conditions at No. 2 Military Hospital, Old Park, Canterbury, which it was alleged is in a wretched condition for the military patients who were brought there, huts of fifty beds having only one orderly in charge, being insufficiently warmed, and the sanitary and lavatory accommodation being 200 yards away. Mr. Macpherson said that orders had been given for this hospital to be closed until alterations which are recommended by the Sanitary Committee can be carried out.

### Three Patients in Two Beds.

MR. HOGGE asked the Under-Secretary of State for War whether the accommodation at Stobhill Hospital is so crowded that two beds are put together and three men placed in them, and whether at Bellahouston patients are placed on the floor. Mr. Macpherson said that he had asked for a report on this matter.

